# Sea ice pattern effect on Earth’s energy budget is characterized by hemispheric asymmetry

**DOI:** 10.1126/sciadv.adr4248

**Published:** 2025-02-28

**Authors:** Chen Zhou, Qingmin Wang, Ivy Tan, Lujun Zhang, Mark D. Zelinka, Minghuai Wang, Jonah Bloch-Johnson

**Affiliations:** ^1^ School of Atmospheric Sciences, Nanjing University, Nanjing, China.; ^2^ Frontiers Science Center for Critical Earth Material Cycling, Nanjing University, Nanjing, China.; ^3^ Department of Atmospheric and Oceanic Sciences, McGill University, Montreal, Canada.; ^4^ Lawrence Livermore National Laboratory, Livermore, CA, USA.; ^5^ Department of Earth and Climate Sciences, Tufts University, Medford, MA, USA.; ^6^ National Center for Atmosphere Science, University of Reading, Reading, UK.

## Abstract

Earth’s energy budget is sensitive to the spatial distribution of sea surface temperature and sea ice concentration (SIC) change, but the global radiative effect of changes in SIC spatial distribution has not been quantified. We show that SIC-induced radiation anomalies at the top of the atmosphere are sensitive to the location of SIC reduction in each season, which qualitatively explains how and why the effect of sea ice loss on Earth’s energy budget is determined by its spatial pattern. Idealized experiments indicate that SIC-induced surface warming is greater in the Arctic regions, resulting in a more negative Planck feedback. Global low-level cloud cover responses to Arctic and Antarctic SIC reduction are also distinct, leading to more negative SIC-cloud feedback in Arctic regions. SIC-induced albedo feedback is sensitive to latitude due to inhomogeneous solar radiation at the surface. As a result, the simulated radiative effect of SIC anomalies during 1980–2019 is dominated by variations in the spatial pattern of SIC.

## Teaser

Effect of sea ice loss on Earth’s energy budget at the top of atmosphere depends on its spatial pattern.

## INTRODUCTION

Global warming is a result of imbalance in the Earth’s energy budget (*1*). In response to changes in greenhouse gases and aerosol concentration, the net radiative flux at the top of atmosphere (TOA) changes due to instantaneous radiative forcing and rapid adjustments, leading to an effective radiative forcing to the Earth’s climate system (*1*, *2*). Sea surface temperature (SST) and sea ice change gradually in response to radiative forcings, and corresponding changes in cloud properties, lapse rate, surface albedo, and humidity further change the Earth’s energy budget and provide feedbacks to the climate system (*1*). The magnitude of climate feedbacks depends on the spatial pattern and magnitude of the effective radiative forcing, leading to differences in forcing efficacies (*3*–*5*). Moreover, climate fluctuations alter the Earth’s energy budget by changing the spatial pattern of SST and sea ice. According to (*6*), the change in global TOA net radiation *N* relative to the preindustrial level can be expressed as

N=F−λΔT+P+ε (1)

where *F* denotes the effective radiative forcing relative to the preindustrial level, Δ*T* is the change of global mean surface temperature, λ is the long-term climate feedback parameter in response to CO_2_ forcing, and *P* is the radiation anomaly induced by the change of SST/sea ice pattern relative to CO_2_-induced long-term global warming [i.e., the pattern effect (*7*–*12*)], and ε is a residual term induced by transient atmospheric processes. λ is a function of Δ*T* due to the state dependence of climate feedbacks (*13*–*15*), which can be approximated as a constant number when Δ*T* is not large (e.g., less than 1°). Land surface temperature responds rapidly to changes in radiative forcings and SST/sea ice, so it is not explicitly expressed in Eq. 1. The importance of the SST pattern effect in the evolution of Earth’s energy budget has been demonstrated in previous studies, and the main mechanisms of how the SST pattern affects the Earth’s energy budget have been revealed (*9*, *11*, *12*, *16*). On the other hand, although the existence of a sea ice concentration (SIC) pattern effect (i.e., the change of global TOA radiative anomaly in response to changes in the spatial distribution of SIC) has been expected (*17*, *18*), its magnitude has not been quantified, and its underlying physical mechanisms have not been fully elucidated.

Observations reveal a global sea ice loss under global warming. The sea ice area and SIC reduction is significant in the Arctic, while there is no significant trend in annual mean Antarctic sea ice area during 1979–2018 (*19*, *20*, *21*).Meanwhile, there is low confidence in the long-term trend of sea ice thickness (*19*, *22*, *23*). The impact of sea ice change on the global climate system is important and complicated (*24*–*27*). During the past several decades, the radiative effect of SIC reduction over polar regions has warmed the Earth system by reducing the Earth’s surface albedo (*19*). Low-level cloud fraction in the Arctic generally increases as the SIC decreases depending on the thermodynamic structure of the atmosphere, and the warming effect of the surface albedo reduction is dampened by the masking effect of clouds, with strong seasonal dependence (*28*–*32*). Surface and air temperatures increase in response to sea ice loss, and the corresponding air temperature feedback also dampens the warming effect of sea ice loss (*33*). The interactions between SIC, temperature, and cloud have been demonstrated to be important in determining the climate of the high latitudes (*28*, *34*–*37*). Differences between Arctic and Antarctic feedbacks have been identified (*38*–*40*), but the pattern effect on SIC-related feedbacks has not been quantified.

In this study, we compare the SIC change patterns in observations and global warming simulations, analyze the climate effect of the SIC pattern effect, and analyze the mechanism how the SIC change pattern affects the Earth’s energy budget using a set of SIC patch experiments.

## RESULTS

### Pattern effect of historical SIC reduction

**Fig. 1.**
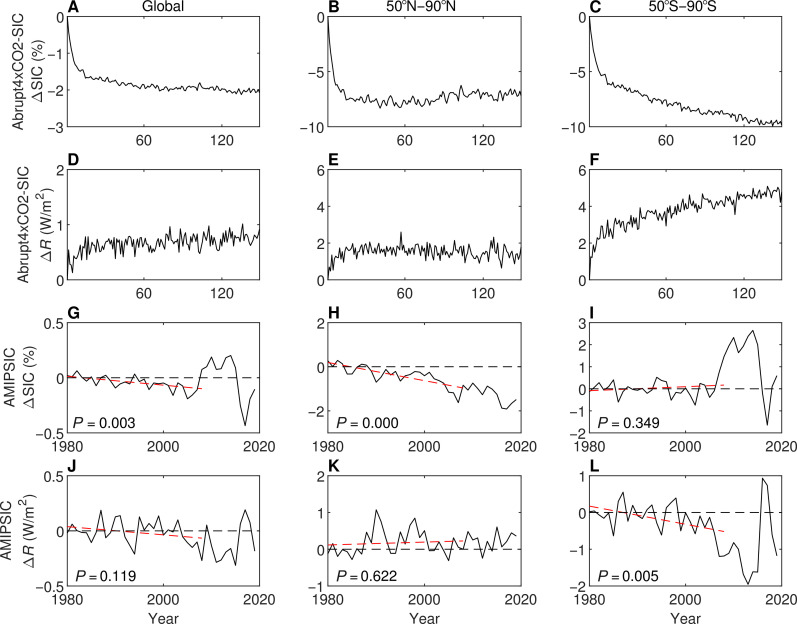
SIC changes and corresponding radiative effects under CO_2_-induced global warming and during the historical period of 1980–2019. (**A** to **F**) Results from the abrupt4xCO2-SIC experiments, where the changes are relative to the first year of the experiment. (**G** to **L**) Results from the AMIPSIC experiments, where the changes are calculated relative to the average value between 1980 and 1989. The red dashed lines denote the trends over 1980–2008, and the *P* values of *t* tests for these trends are shown in each panel. The black dashed lines are base lines.

**Fig. 2.**
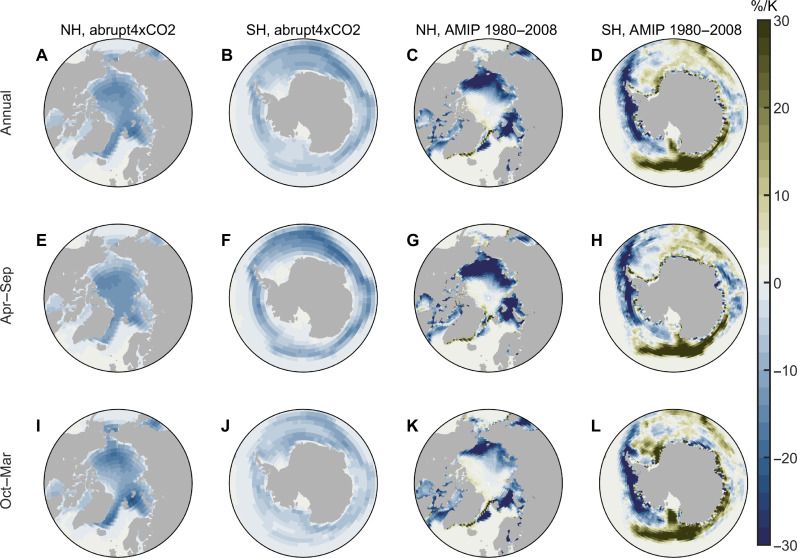
Change of SIC normalized by the change of global mean surface temperature. (**A** and **B**) Annual SIC change pattern in abrupt4xCO2 experiments, calculated using the differences between average value of the piControl experiments and years 131 to 150 of the abrupt4xCO2 experiments (table S1). (**C** and **D**) Historical annual SIC change pattern during 1980–2008 (AMIP SIC). (**E** to **H**) SIC change patterns in April to September. (**I** to **L**) SIC change patterns in October to March.

The spatial pattern of observed SIC trend during the past several decades is different from the SIC change pattern under CO_2_-induced long-term global warming. Under CO_2_-induced global warming, both Arctic and Antarctic SIC decreases in the long-term (Fig. 1, A to C, the abrupt4xCO2 experiment is described in table S1), and the SIC change is negative over almost all grid cells covered by sea ice (Fig. 2, A and B). SIC reduction in the northern hemisphere (NH) primarily occurs during the first 20 years of the abrupt4xCO2 experiments and then stops as SIC approaches zero in August to October. In contrast, SIC in the southern hemisphere (SH) decreases steadily during the whole simulation period, albeit more rapidly in the first two decades after quadrupling. To quantify the radiative effect of SIC in the abrupt4xCO2 experiment, we design a set of abrupt4xCO2-SIC experiments with three individual atmosphere-only simulations driven by SIC of abrupt4xCO2 experiments and repeating climatological monthly mean SSTs from piControl experiments (table S1 and fig. S1). The total SIC-induced radiation change under CO_2_-induced warming is positive in our simulations (Fig. 1, D to F), which is consistent with the expectation that the radiative effect of CO_2_-induced SIC reduction warms the Earth system. The warming effect of SIC reduction is primarily induced by the albedo feedback, whereby sea ice loss allows more solar radiation to be absorbed by the ocean surface (fig. S2). In addition, the surface temperature, lapse rate, humidity, and cloud properties also change in response to SIC reduction, leading to additional feedbacks to the climate system (fig. S2).

The global SIC is also decreasing in the historical period (Fig. 1G). To evaluate the SIC pattern effect on the global energy budget, we perform a set of idealized experiments (AMIPSIC, where AMIP denotes experiment design similar to that in the Atmospheric Model Intercomparison Project), where the radiative forcings and SST are fixed at preindustrial levels, and the SIC are prescribed from observations (see Materials and Methods). The radiative effect of SIC and SST approximately equals the superposition of the SIC radiative effect and the SST effect (fig. S3), so the idealized SIC experiments are valid to quantify the radiative effect of SIC on Earth’s energy budget. In both hemispheres, radiation anomalies induced by a SIC reduction are positive, consistent with abrupt4xCO2-SIC experiments. Unexpectedly, the trend of global ∆*R* is negative despite the statistically significant decrease of global SIC between 1980 and 2008, implying that global SIC reduction leads to planetary cooling during this period (Fig. 1, G and J, and fig. S4). This seemingly counterintuitive result can be better understood by considering the unevenly distributed SIC reduction pattern (Fig. 2). During this period, Antarctic SIC increases over most regions, leading to a decrease in ∆*R* averaged over the SH high latitude regions, while the Arctic SIC generally decreases, leading to an increase in ∆*R* averaged over the NH high latitude regions. The sensitivity of NH Δ*R* to NH Δ*SIC* is smaller than the sensitivity of SH Δ*R* to SH Δ*SIC* (fig. S5), so the radiative cooling induced by Antarctic SIC growth is greater than the radiative heating induced by Arctic SIC reduction. As a result, the relationship between global SIC trend and Δ*R* trend is opposite from that under long-term global warming, and the sea ice pattern effect is important in determining the climate effect of sea ice cover changes.

**Fig. 3.**
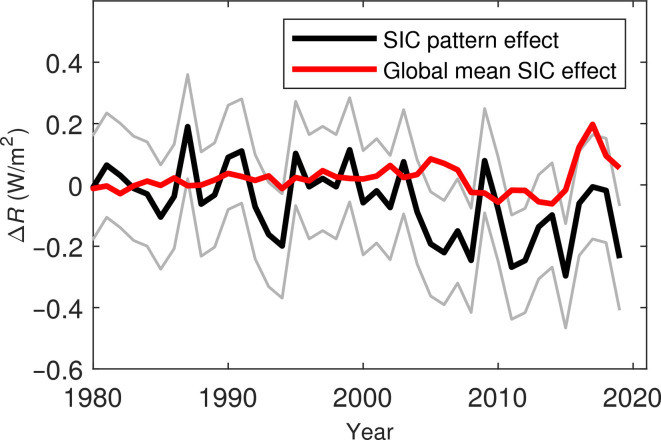
Decomposition of SIC-induced radiation anomalies at TOA. The red line is the radiative effect of global mean SIC anomalies (λ_SIC_Δ*SIC*), and the black line is the SIC pattern effect (*P*_SIC_; see Eq. 7 in Materials and Methods). The gray lines denote the SD interval of the SIC pattern effect.

We quantify the contribution of global SIC radiative effect and SIC pattern effect to the global TOA radiation anomalies (Fig. 3 and see Materials and Methods). The variance of SIC pattern effect (0.0032 W^2^/m^4^) is greater than that of the global SIC radiative effect (0.0024 W^2^/m^4^), indicating that the radiative effect of SIC anomalies during this period is primarily affected by the SIC pattern effect.

### Mechanism of SIC pattern effect

**Fig. 4.**
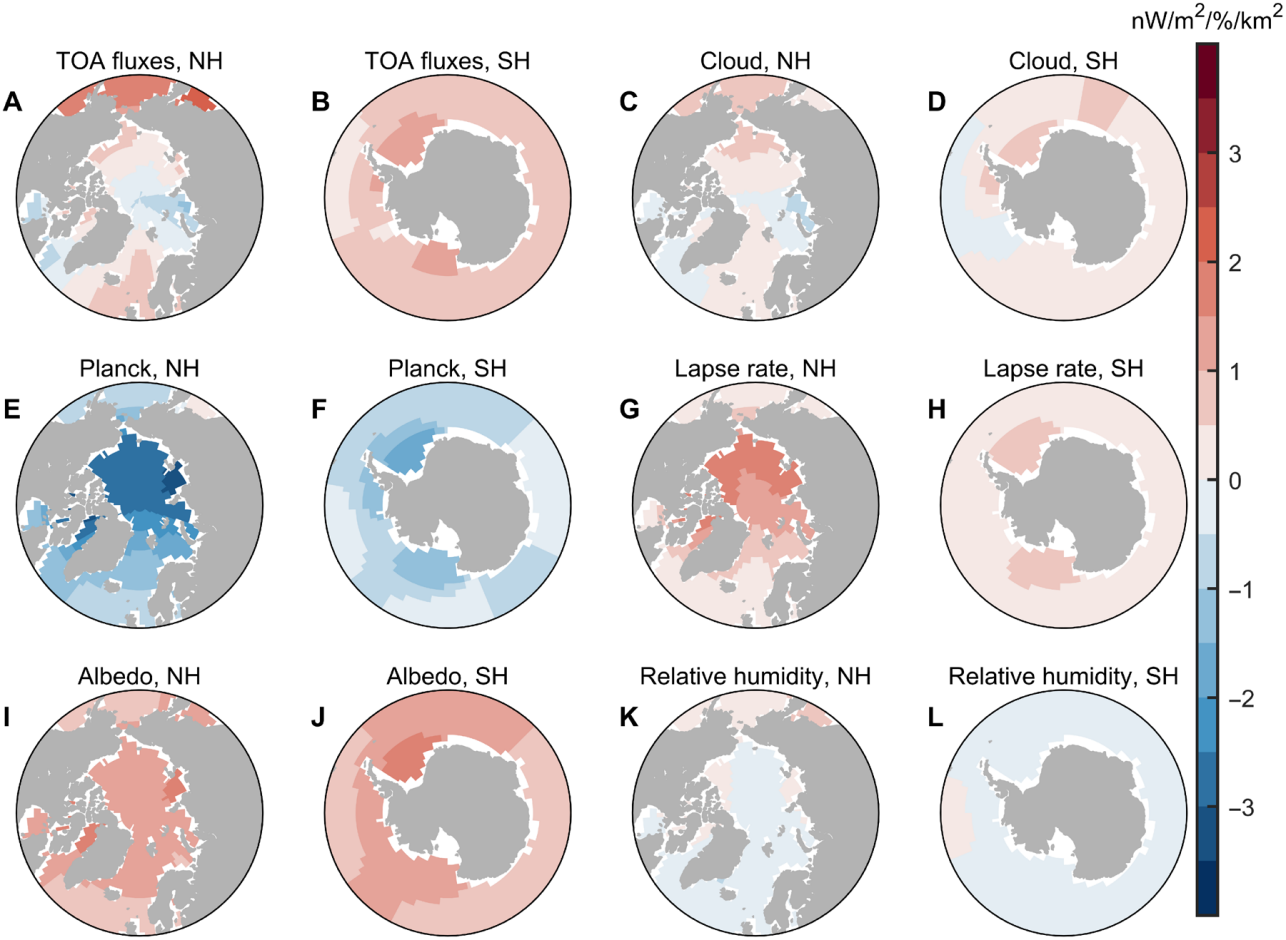
Sensitivity of global TOA radiation to regional SIC reduction and its breakdown into contributions from individual radiative processes. (**A** and **B**) Response of annual mean global TOA radiation to regional SIC reduction in unit surface area (−∂R/∂SIC, calculated with Eq. 4). (**C** and **D**) Contribution of cloud changes. (**E** and **F**) Contribution of vertically uniform temperature changes (Planck feedback). (**G** and **H**) Contribution of vertically nonuniform temperature changes (lapse rate feedback). (**I** and **J**) Contribution of surface albedo changes. (**K** and **L**) Contribution of RH changes. A positive value at a given location indicates that sea ice loss at that location leads to a radiative heating of the climate system, averaged annually and over the globe. Seasonal values and regional responses are shown in figs. S10 to S12.

We investigate the mechanism of the SIC pattern effect by performing a set of sea ice patch perturbation experiments, where the SIC is both increased and decreased within individual patches, and the response of the global radiative effect to regional SIC changes can be calculated (see Materials and Methods and fig. S6). The results show that the magnitude and even the sign of the response of TOA radiative fluxes to regional SIC reductions are sensitive to the location of SIC change (Fig. 4, A and B). The responses of TOA radiative fluxes to regional SIC reductions are further decomposed into contributions from changes in surface temperature (Planck feedback), lapse rate, relative humidity (RH), cloud, and surface albedo (see Materials and Methods) (*41*).

The primary contributor to the hemispheric asymmetry is the Planck feedback [Fig. 4, E and F; fixed RH framework (*41*)]. When SIC decreases in the Arctic region, surface temperature in the Arctic regions increases (*33*), leading to more longwave emission to space (i.e., stronger negative Planck feedback). The radiative effect of the Planck feedback is partially counteracted by the lapse rate feedback (Fig. 4, G and H). In comparison, surface warming in response to Antarctic SIC reduction is weaker, so the cooling effect from the Planck feedback is weaker in response to SIC reduction in most Antarctic regions. In addition, the lapse rate feedback is also weaker in the Antarctic regions (*42*).

Moreover, responses of cloud radiative effect (CRE) to SIC reduction are also distinct in the two hemispheres. The mechanisms behind the SIC-induced cloud radiation anomalies are complex, and part of the hemispheric asymmetry is induced by the change of low-level cloud cover (LCC; cloud fraction with cloud top pressure greater than 680 hPa; figs. S7 and S8). When the sea ice melts, the saturation water vapor pressure of the air above the sea surface increases, so more water vapor is able to enter the atmosphere. Meanwhile, the surface air temperature increases in response to SIC reduction, leading to a decrease in local near-surface static stability and estimated inversion strength (EIS) (*43*), which have competing effects on LCC. Decreasing near-surface static stability favors boundary layer moistening and an increase in LCC (*28*), while the simultaneous decrease of EIS (*43*) and increase of surface temperature (*44*) favor a decrease in LCC. The effect of water vapor and near-surface stability dominates over the effect of surface temperature and EIS inside the Arctic and Antarctic circles, where climatological SIC and EIS are high and surface water vapor concentration is low [fig. S8, consistent to (*28*)]. In contrast, the surface temperature and EIS effects on LCC tend to be more important over lower latitudes. As a result, the LCC increases notably in response to SIC reduction in most Arctic regions but does not increase as much in response to SIC reduction in most Southern Ocean areas (figs. S7 and S8), contributing to the hemispheric asymmetry on cloud-SIC feedback. The different Arctic and Antarctic SIC-LCC relationship implied by the idealized simulations is generally consistent with Moderate Resolution Imaging Spectroradiometer (MODIS) satellite observations (fig. S9).

The response of global TOA fluxes to SIC reduction depends on the season. There is little solar radiation in wintertime, so the surface albedo–induced radiation anomalies are greater during summertime. Moreover, SIC-induced surface warming is stronger in wintertime, leading to a stronger negative Planck feedback in wintertime (figs. S10, E and F, and S11, E and F). This asymmetry arises because the ice surface temperature is colder in wintertime, while the water surface temperature is close to the ice melting point for all seasons, so the local temperature change induced by SIC reduction is also greater in wintertime. As a result, the SIC-induced radiation anomalies tend to cool Earth in wintertime and warm Earth in summertime for both hemispheres (figs. S10 and S11). It is also worth noting that most SIC-induced TOA radiation anomalies are contributed from high latitudes of the corresponding hemisphere (fig. S12).

As a result, SIC reduction over the SH leads to a radiative heating on the Earth’s climate system, but SIC reduction near the North Pole leads to a radiative cooling. The above mechanism could partially explain how the SIC-induced radiation anomalies vary during the recent decades (fig. S13A). Note that although the sensitivity of global TOA fluxes to SIC reduction in central Arctic Ocean is negative (Fig. 4), the SIC trend over these regions is small, so the contribution of the central Arctic Ocean to the trend of TOA fluxes is negligible; instead, the negative trend during this period is primarily induced by the SIC increase in the Antarctic regions.

## DISCUSSION

Our findings show that the global climate effect of sea ice loss depends on its spatial pattern, and the SIC pattern effect is characterized by hemispheric asymmetry. SIC reduction in Arctic regions induces greater surface warming and a correspondingly greater radiative cooling effect due to the Planck feedback than the Antarctic regions. Cloud radiative effect changes are typically more positive when SIC reductions occur over lower latitude regions with smaller mean-state SIC and weaker lower tropospheric stability. SIC-induced albedo feedback is sensitive to latitude due to inhomogeneous solar radiation at the surface. As a result, numerical simulations indicate that the bulk radiative effect of SIC reduction with certain spatial patterns (e.g., trends during 1980–2008) could even cool Earth due to the hemispheric asymmetry of SIC change.

Considering that historical SIC varies across different datasets (*45*), we reperform the AMIPSIC experiment with SIC from Hadley Centre sea ice and SST dataset (HadISST) (*46*) and National Snow and Ice Data Center (NSIDC) (*47*). The trend of TOA fluxes during 1980–2008 is also negative when HadISST is used (fig. S14), despite different statistical metrics. When SIC from NSIDC is used, the TOA fluxes trend during 1980–2008 is close to zero (fig. S14), but the variance of SIC pattern effect (0.0086 W^2^/m^4^) is also greater than that of the global SIC radiative effect (0.0027 W^2^/m^4^) during the past four decades, so the SIC pattern effect is still important.

The SIC pattern effect simulated by climate models is also affected by model uncertainty. To assess the model dependence of the SIC pattern effect, we performed the AMIPSIC experiment with another model [HadAM3 (*48*)], and the results show similar hemispheric asymmetry of Δ*R* sensitivity to SIC reduction, despite differences in the magnitude of the pattern effect (fig. S15). In the future, the intermodel spread of SIC pattern effect might be quantified under the framework of the Green’s Function Model Intercomparison Project (*49*).

The radiative effect of sea ice thinning is not analyzed in this study due to lack of reliable global long-term sea ice thickness observations (*22*). Changes in sea ice thickness can affect the Earth’s energy budget by altering the surface albedo, and it is likely that the climate effects of sea ice thinning also depend on its spatial pattern due to unevenly distributed solar irradiance and regional variations in mean-state sea ice thickness. This potential sea ice thickness pattern effect might be analyzed in future works.

## MATERIALS AND METHODS

### Historical simulation

Here, the simulations are performed using the Community Earth System Model 1.2.1 with Community Atmosphere Model 5.3 (CESM1.2.1-CAM5.3) at 1.9 latitude × 2.5 longitude resolution (*50*). The effective climate sensitivity of this model version is ~3.0 K, which is close to the optimal estimation of the Sixth Assessment Report of Intergovernmental Panel on Climate Change (IPCC AR6) (*1*). In prescribed SST/SIC experiments, sea ice thickness is set to be fixed in each hemisphere by the model, and the default shortwave radiative transfer scheme is used in the sea ice model component. The radiative effect of sea ice thinning is not analyzed in this study.

A set of AMIP-like simulations are carried out to test whether the SST-induced radiation anomalies and SIC-induced radiation anomalies superpose linearly. These simulations include an AMIPFF experiment with historical SST/SIC and fixed radiative forcing (fixed at preindustrial levels in this study), an AMIPSST experiment with historical SST and fixed SIC/forcing, and an AMIPSIC experiment with historical SIC and fixed SST/forcing (table S1). The historical SST/SIC datasets of (*51*) are used in these AMIP-like simulations and are from the CESM website. This AMIP SST/SIC dataset is a merged product based on HadISST and version 2 of the National Oceanic and Atmospheric Administration (NOAA) weekly optimum interpolation SST analysis and provides monthly mean SST and SIC data from 1870 to the present. Each AMIP experiment has three individual ensemble members, and each individual ensemble is performed with slightly perturbed initial conditions.

We also perform the AMIPSIC experiment with HadISST and NOAA/NSIDC climate data record of passive microwave sea ice concentration (denoted as NSIDC SIC in this study) datasets. The sea ice data of HadISST are obtained from a variety of data sources including digitized sea ice charts derived from shipping, expeditions, and other activities and microwave-based satellite retrievals and cover the period from 1870 to present. The comprehensive integration of diverse data sources provides a long-term perspective on sea ice trends, making HadISST suitable for historical climate analyses and as boundary conditions for climate models. The NSIDC SIC data provide satellite-based SIC observations from 1978 to present, using passive microwave sensors such as Scanning Multichannel Microwave Radiometer (SMMR), Special Sensor Microwave/Imager (SSM/I) and Special Sensor Microwave Imager/Sounder (SSMIS) across various satellite platforms. The temporal coverage of AMIP SST/SIC data and HadISST data are about 100 years longer than that of NSIDC SIC data, and they are more frequently used in global climate simulations and related studies.

### SIC patch experiments

A series of SIC patch experiments have been carried out to analyze the effect of regional SIC change on the global climate system, which can be used to quantify the effect of changes in SIC spatial distribution.

Twenty-seven patches are used to cover the ocean area of the high latitudes (fig. S6). In each SIC patch experiment, the SIC of a specific patch is changed using the following equation

$$\begin{array}{c} \Delta {\mathrm{SIC}}_{p,\text{ice}}\left(\mathrm{lat},\mathrm{lon}\right)=A{\text{cos}}^{2}\left(\frac{\pi }{2}\frac{\mathrm{lat}-{\mathrm{lat}}_{p}}{{\mathrm{lat}}_{w}}\right){\text{cos}}^{2}\left(\frac{\pi }{2}\frac{\mathrm{lon}-{\mathrm{lon}}_{p}}{{\mathrm{lon}}_{w}}\right)\\ \left(\left|\mathrm{lat}-{\mathrm{lat}}_{p}\right|<{\mathrm{lat}}_{w},\left|\mathrm{lon}-{\mathrm{lon}}_{p}\right|<{\mathrm{lon}}_{w}\right) \end{array}$$ (2)

where *A* is set to be +0.4 and −0.4 in conjugate SIC increase and reduction experiments in this study; *lat_p_* and *lon_p_* are the latitude and longitude of the center point for the *p*th patch, respectively; *lat_w_* and *lon_w_* are the meridional and zonal half width of the patch, respectively. This equation is similar to the equations for SST patch experiments (*11*, *12*, *52*). If SIC is greater than 100% after the above equation is applied, then it is set to be 100%; SIC is set to be zero if its value is negative.

Then, the sensitivity of global radiative flux to regional SIC change in a specific grid box (*i*th grid box) can be calculated as (*11*)

$$\frac{\partial R}{\partial {\mathrm{SIC}}_{i}}=\frac{\sum_{p}\left(R_{p,\text{ice}+}-R_{p,\text{ice}-}\right)\frac{\left(SIC_{p,i,\text{ice}+}-SIC_{p,i,\text{ice}-}\right)}{\left(SIC_{p,\text{avg},\text{ice}+}-SIC_{p,\text{avg},\text{ice}-}\right)}\frac{S_{i}}{S_{p}}}{\sum_{p}\left(SIC_{p,i,\text{ice}+}-SIC_{p,i,\text{ice}-}\right)}$$ (3)

where *p* denotes the perturbation experiments performed in the *p*th patch, $R_{p,\text{ice}+}$ and $R_{p,\text{ice}-}$ are the time-average (all data are used to calculate annual sensitivities, and only data of a specific month are used to calculate monthly resolved sensitivities) radiative fluxes in the conjugate SIC increase and reduction experiments, respectively, ${\mathrm{SIC}}_{p,i,\text{ice}+}$ and ${\mathrm{SIC}}_{p,i,\text{ice}-}$ are the SIC in the *i*th grid box in conjugate SIC increase and reduction experiments, respectively, ${\mathrm{SIC}}_{p,\text{avg},\text{ice}+}$ denotes the average SIC in the *p*th patch, *S_i_* is the ocean surface area of the *i*th grid box, and *S_p_* is the ocean surface area of the *p*th patch. The radiative fluxes are integrated over the full spectral range (10 to 50000 cm^−1^), and downward radiation is defined to be positive in this study.

Near the poles, the sensitivity calculated from Eq. 3 is small due to the relatively small area of each grid box (the area of each grid box is proportional to the cosine of latitude). To avoid the effect of nonuniform grid box area, the sensitivity of global radiative flux to regional SIC change is divided by the surface area of each grid box

$$\frac{\partial R}{\partial SIC}=\frac{\partial R}{\partial SIC_{i}}\frac{1}{S_{i}}$$ (4)

These normalized sensitivities are shown in Fig. 4.

Although the radiative effects of SIC change are strongly nonlinear, the Green’s function approach largely captures the global SIC-induced radiation anomalies in AMIPSIC simulations (fig. S13A). The Green’s function approach underestimates the SIC-induced radiation change in abrupt4xCO2-SIC simulations, but the correlation is still high (fig. S13B), so it is valid for attribution analyses.

Considering that SST at each grid box might increase simultaneously when SIC reduces, we perform an additional set of SIC-SST covarying patch experiments. The SIC-SST covarying patch experiments are similar to the SIC patch experiments, except that SST in a specific grid is changed proportionally with SIC

$$\text{Δ}SST_{p,\text{icesst}}\left(\mathrm{lat},\mathrm{lon}\right)=B\Delta SIC_{p,\text{icesst}}\left(\mathrm{lat},\mathrm{lon}\right)$$ (5)

where *B* is a constant and is set to be −0.05 K/% here (note that the relationship between Δ*SST* and Δ*SIC* is nonlinear in reality). The sensitivities of radiation anomalies to regional SIC changes calculated from SIC-SST patch experiments are similar to that calculated from SIC patch experiments (fig. S16).

### Quantification of SIC pattern effect

The SIC-induced radiation anomalies can be decomposed into a global SIC component and a SIC pattern effect term

$$\Delta R_{\text{SIC}}=\lambda_{\text{SIC}}\text{Δ}SIC+P_{\text{SIC}}+\epsilon$$ (6)

where λ_SIC_ is the regression slope of global Δ*R* against global Δ*SIC* in the abrubpt4xCO2 experiment, $P_{\text{SIC}}$ is the SIC pattern effect, and ϵ is an error term that is induced by factors other than SIC. The variance of ϵ in an individual run [var(ϵ)] can be calculated from the differences between individual AMIPSIC runs. Then, the pattern effect $P_{\text{SIC}}$ can be estimated as

$${\hat{P}}_{\text{SIC}}=\Delta R_{\text{SIC}}-\lambda_{\text{SIC}}\text{Δ}SIC$$ (7)

The variance of estimated ${\hat{P}}_{\text{SIC}}$ is greater than the variance of actual $P_{\text{SIC}}$, due to the error term in Eq. 6. Assuming that ϵ is independent of other terms, the variance of $P_{\text{SIC}}$ in an individual run can be calculated as

$$\text{var}\left(P_{\text{SIC}}\right)=\text{var}\left(\Delta R_{\text{SIC}}-\lambda_{\text{SIC}}\Delta SIC\right)-\text{var}\left(\epsilon \right)$$ (8)

### Decomposition of radiation anomalies using radiative kernels

Radiative kernels for CESM1 (*53*) are used to decompose the radiation anomalies in this study using the methods of (*54*). Radiation anomalies induced by noncloud perturbations are calculated directly using the following equation

$$\Delta R_{X}=K_{X}\Delta X$$ (9)

where Δ*X* denotes the monthly anomaly of a specific noncloud variable (albedo, surface temperature, air temperature, and humidity), and *K_X_* is the corresponding radiative kernel (*53*, *54*). The humidity-induced radiation anomalies are divided into a component induced by temperature change assuming fixed RH (Δ*R*_Ta_RH_) and a component induced by changes in RH (Δ*R*_RH_) following (*41*), and Δ*R*_Ta_RH_ is subsequently decomposed into a Planck term (Δ*R*_Planck_, change of radiation anomalies assuming that the air temperature change is the same as the surface temperature change) and a lapse rate term (Δ*R*_LR_). Cloud-induced radiation anomalies are computed using the adjusted CRE method

$$\Delta R_{\text{cloud}}=\Delta \text{CRE}-\sum_{X}\left(K_{X}-K_{X}^{0}\right)\Delta X$$ (10)

where CRE is calculated as the difference between all-sky and clear-sky TOA radiative fluxes, and $K_{X}^{0}$ is radiative kernels for clear-sky fluxes.
