# Supplementary material for: Sea ice pattern effect on Earth’s energy budget is characterized by hemispheric asymmetry
Source: Sci Adv. 2025 Feb 28;11(9):eadr4248. doi: 10.1126/sciadv.adr4248 (PMC11870058; doi:10.1126/sciadv.adr4248)
Supplement: Supplementary file 1 — Figs. S1 to S16 Table S1 [file sciadv.adr4248_sm.pdf]

Science Advances
AAAS

Supplementary Materials for

Sea ice pattern effect on Earth's energy budget is characterized by hemispheric asymmetry

Chen Zhou *et al.*

Corresponding author: Chen Zhou, czhou17@nju.edu.cn

Sci. Adv. **11**, eadr4248 (2025)
DOI: 10.1126/sciadv.adr4248

This PDF file includes:

Figs. S1 to S16
Table S1

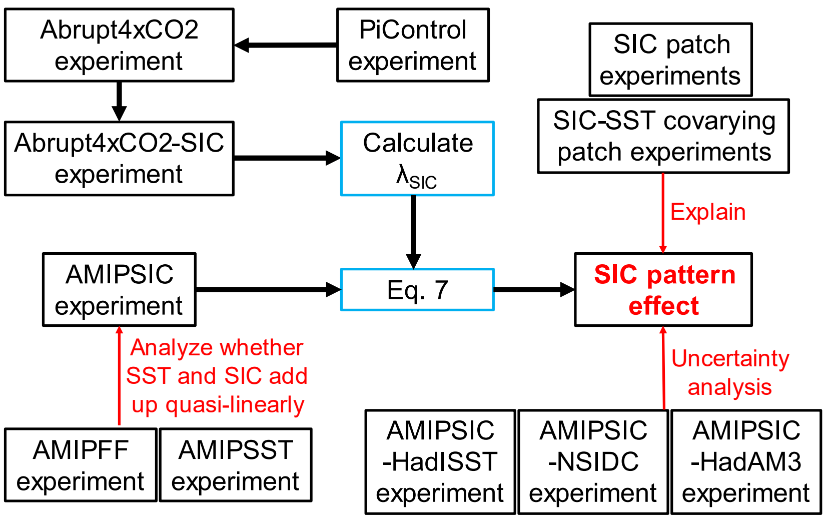

Fig. S1.

Flow chart of idealized experiments that are used to analyze SIC pattern effect.

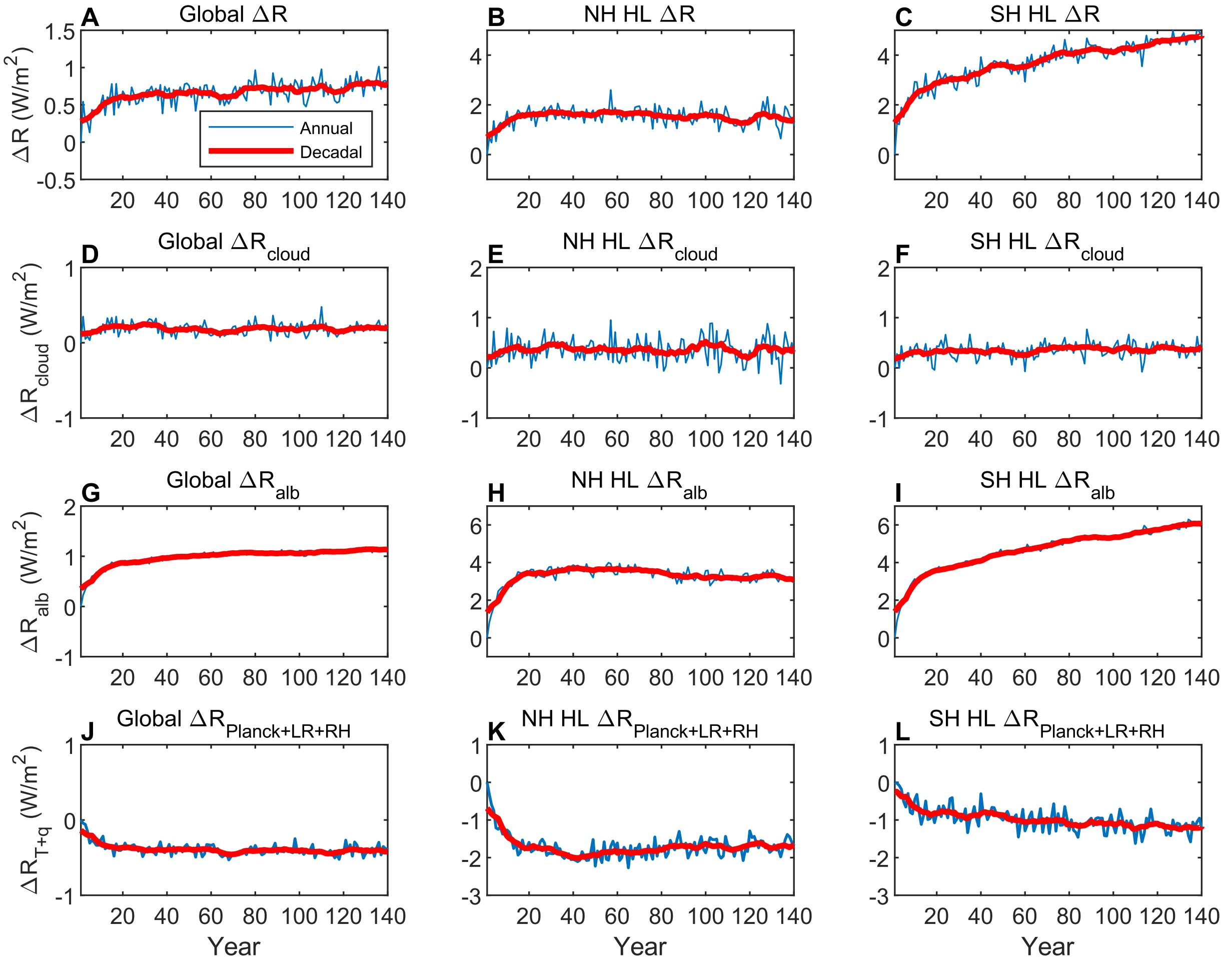

Fig. S2.

TOA radiative change in the abrupt4xCO2-SIC experiments, where the first year is used as the base year. (A-C) Net TOA fluxes. (D-F) Cloud-induced radiation anomalies. (G-I) Albedo-induced radiation anomalies. (J-L) Temperature and water vapor-induced radiation anomalies (Planck + Lapse rate + RH feedbacks). Left column is for global, middle column is for Northern Hemisphere (NH) high latitudes (HL), 50°N-90°N, and right column is for Southern Hemisphere (SH) HL, 50°S-90°S. Note that Δ*R* in SH high latitudes is also affected by SIC changes in NH, and vice versa.

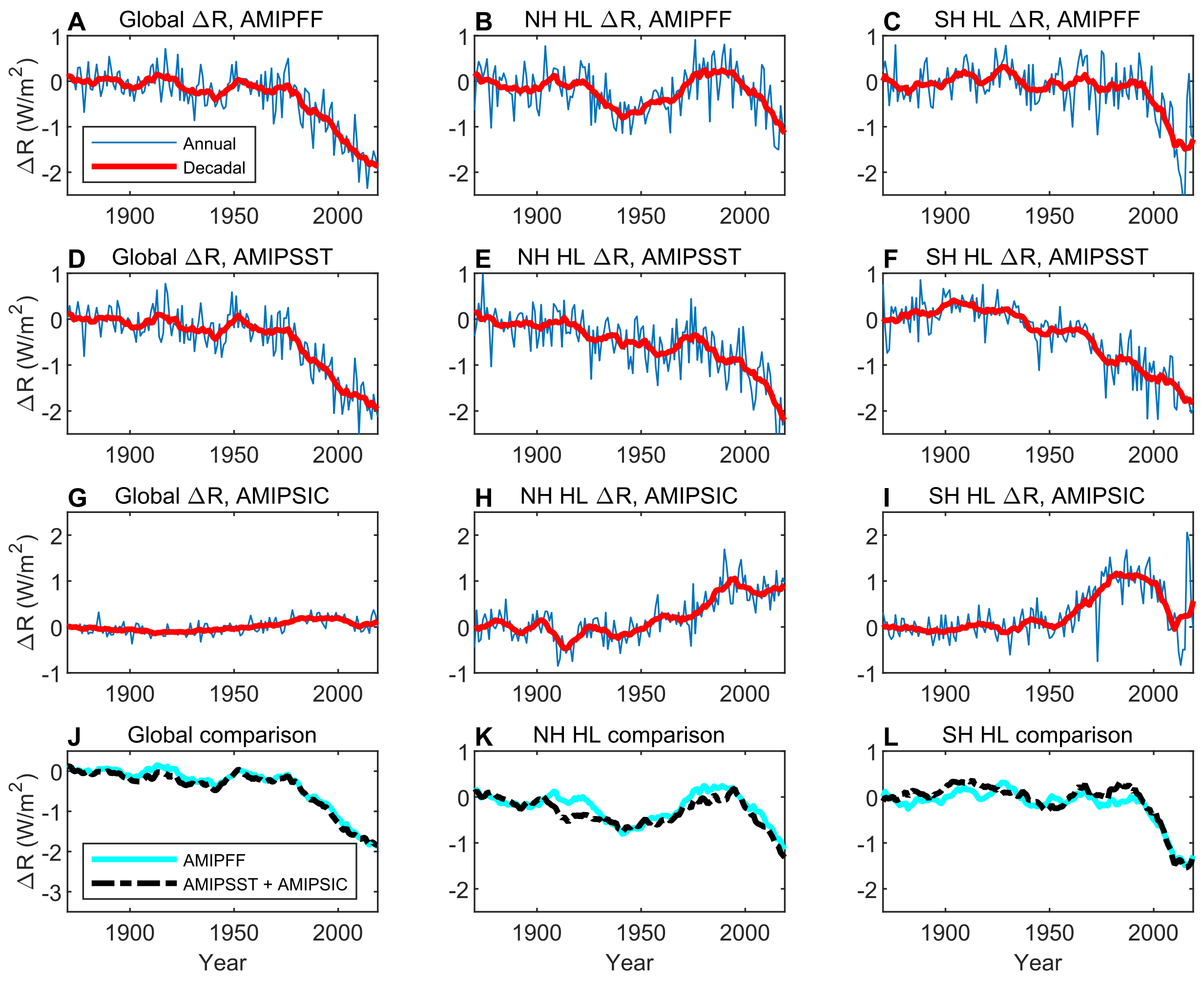

Fig. S3.

Linearity check for the superposition of SST-induced and SIC-induced TOA radiation anomalies. (A-C) Change of TOA net radiation anomalies in AMIPFF experiments. (D-F) Change of TOA net radiation anomalies in AMIPSST experiments, where SST is same as observation and other variables are set to be fixed. (G-I) Change of TOA net radiation anomalies in AMIPSIC experiments, where SIC is same as observation and other variables are set to be fixed. (J-L) Comparison of radiation anomalies in AMIPFF experiments (cyan lines) and AMIPSST+AMIPSIC experiments (black dashed lines). The left column is for global mean radiation anomalies, middle column is for mean radiation anomalies in Northern hemisphere (NH) high latitudes (HL, 50°S-90°S), and the right column is for Southern Hemisphere (SH) high latitudes (50°S-90°S). The results suggest that the radiation anomalies induced by SST/SIC changes can be decomposed to SST-induced component and SIC-induced component using AMIPSIC and AMIPSST experiments.

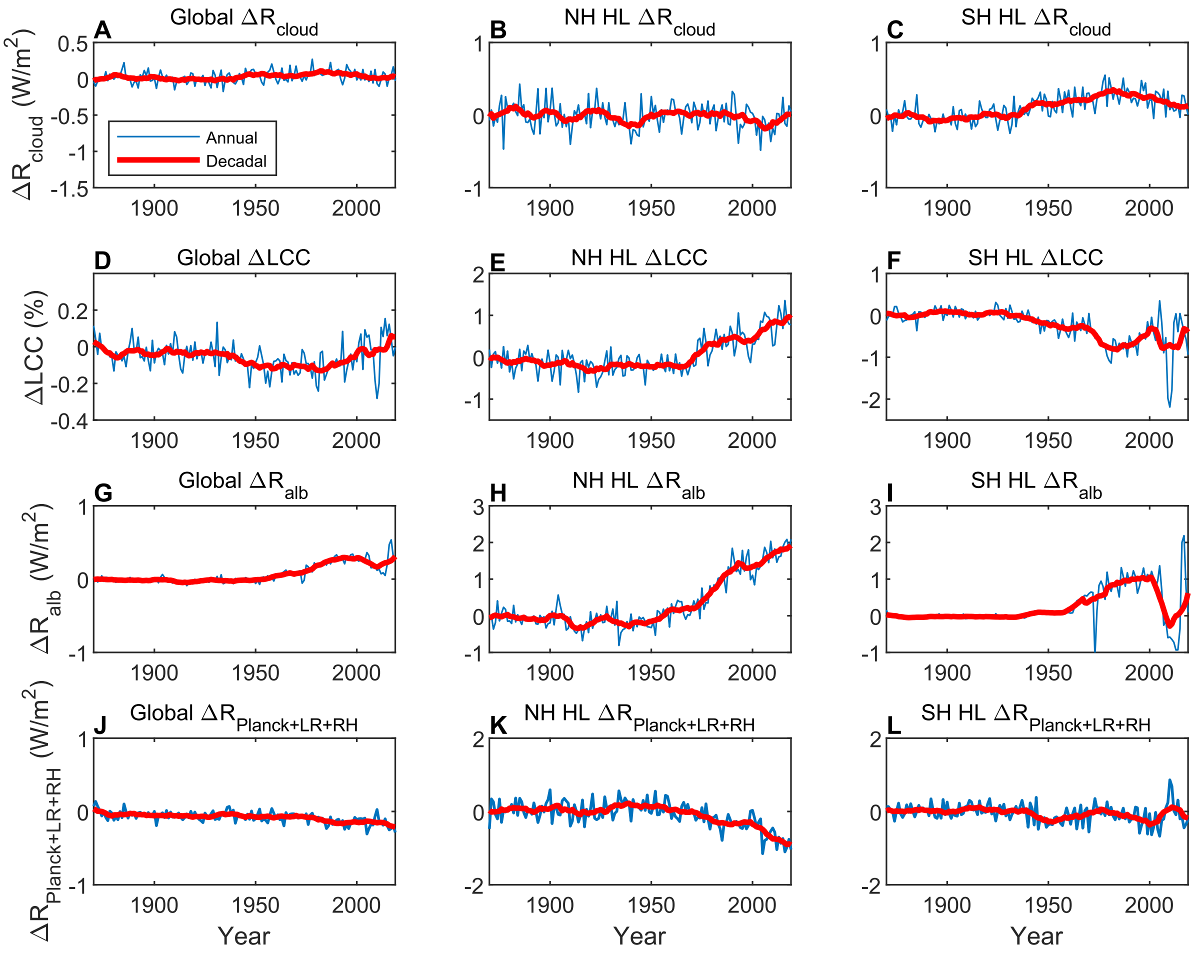

Fig. S4.

Individual radiative effect and low-level cloud cover (LCC) anomalies in AMIPSIC experiments. (A-C) Cloud radiative effects (masking effects are removed). (D-F) LCC changes. (G-I) Albedo-induced radiation anomalies. (J-L) Temperature and water vapor-induced radiation anomalies (Planck + Lapse rate + RH). Left column is for global, middle column is for 50°N-90°N, and right column is for 50°S-90°S. The correlation coefficients between annual ΔR_{cloud} and ΔLCC are -0.43, -0.33, and -0.58 for global, NH HL and SH HL, respectively, and the correlation coefficients between decadal (11-year smoothed) ΔR_{cloud} and ΔLCC are -0.73, -0.36 and -0.87 for global, NH HL and SH HL, respectively. These results suggest that changes in LCC are important to ΔR_{cloud}, while other factors (e. g., the masking effect of low clouds over sea ice) are also important.

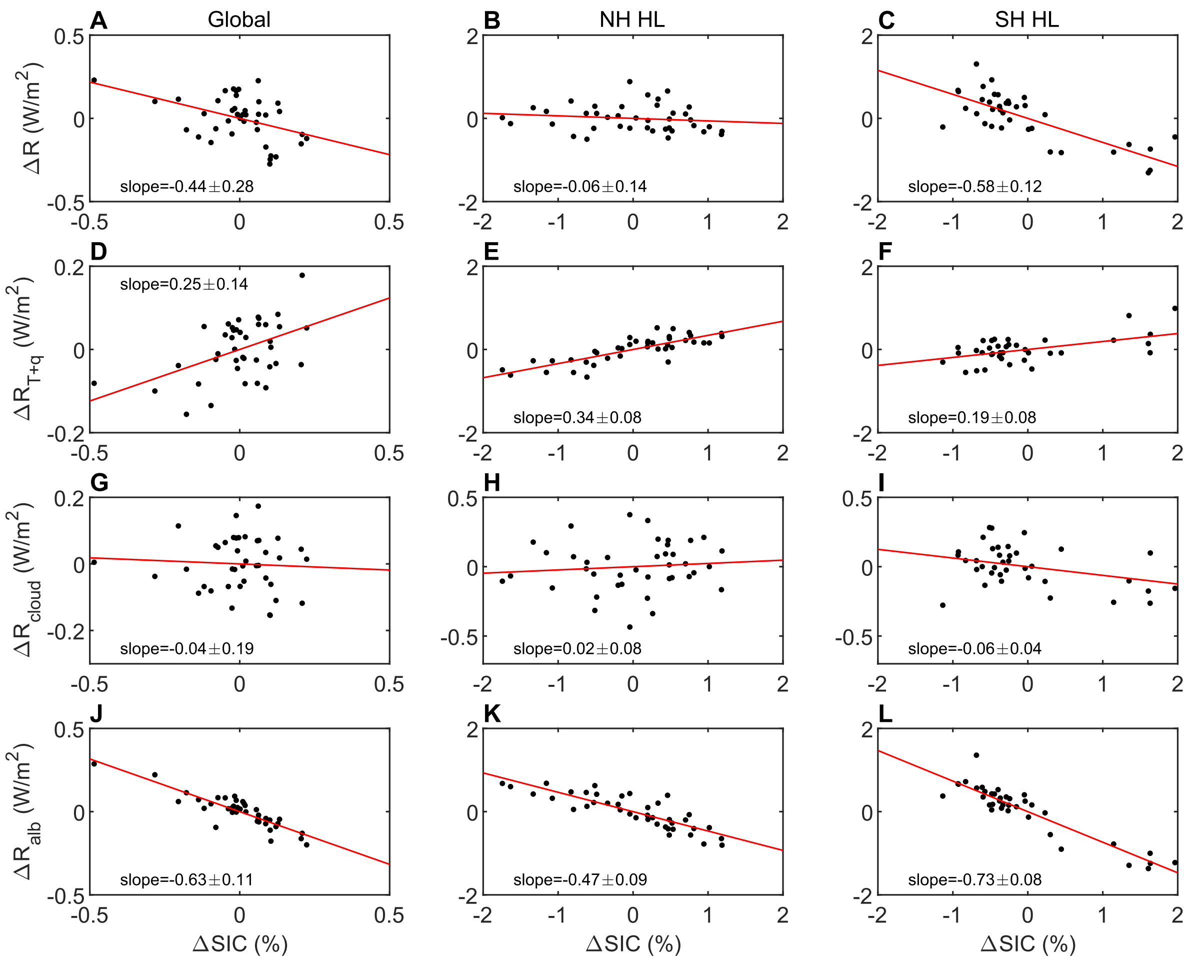

Fig. S5.

Relationship between SIC anomalies and radiation anomalies in the AMIPSIC experiment during 1980-2019. The first row shows the relationship between the annual anomalies of SIC and TOA net radiation, the second row shows the relationship between SIC anomalies and temperature + water vapor-induced radiation anomalies (Planck + Lapse rate + RH), the third row shows the relationship between SIC anomalies and cloud-induced radiation anomalies, and the last row shows the relationship between SIC anomalies and albedo-induced radiation anomalies. Left column is global mean values, middle column is for values averaged over 50°N-90°N, and the right column is for 50°S-90°S. The results suggest that the sensitivity of NH Δ*R* to NH SIC (the change of Δ*R* in response to unit SIC change) is smaller than the sensitivity of SH Δ*R* to SH SIC during this period. The regression slope is shown in each panel, and the 95% uncertainty intervals are also displayed.

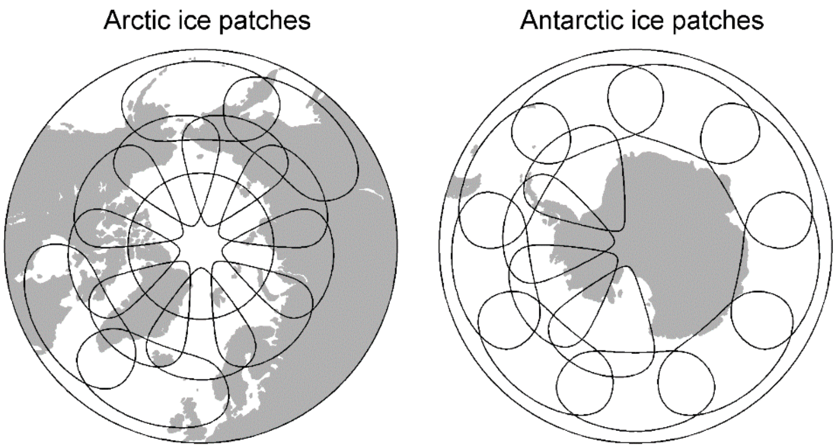

Fig. S6.

Illustration of patches used in the patch experiments. Each circle illustrates the geolocation of a specific patch. The patch size is chosen to match the SST patch in ref. (*11*).

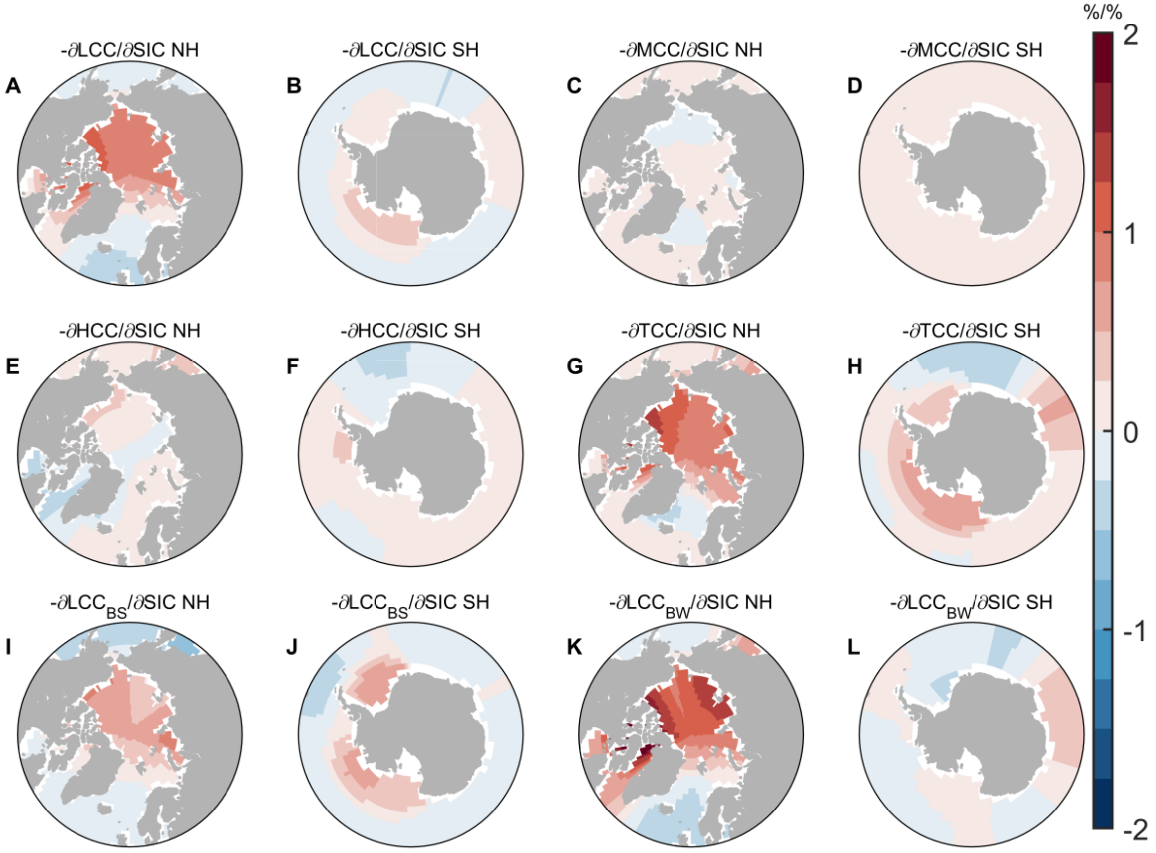

Fig. S7.

Sensitivity of global mean cloud cover to regional SIC reduction, normalized by the surface area of each grid box. (A-B) Annual low-level cloud cover (LCC) responses. (C-D) Annual middle-level cloud cover (MCC) responses. (E-F) Annual high-level cloud cover (HCC) responses. (G-H) Annual total cloud cover (TCC) responses. (I-J) LCC responses during April-September (boreal summer). (K-L) LCC responses during October-March (boreal winter).

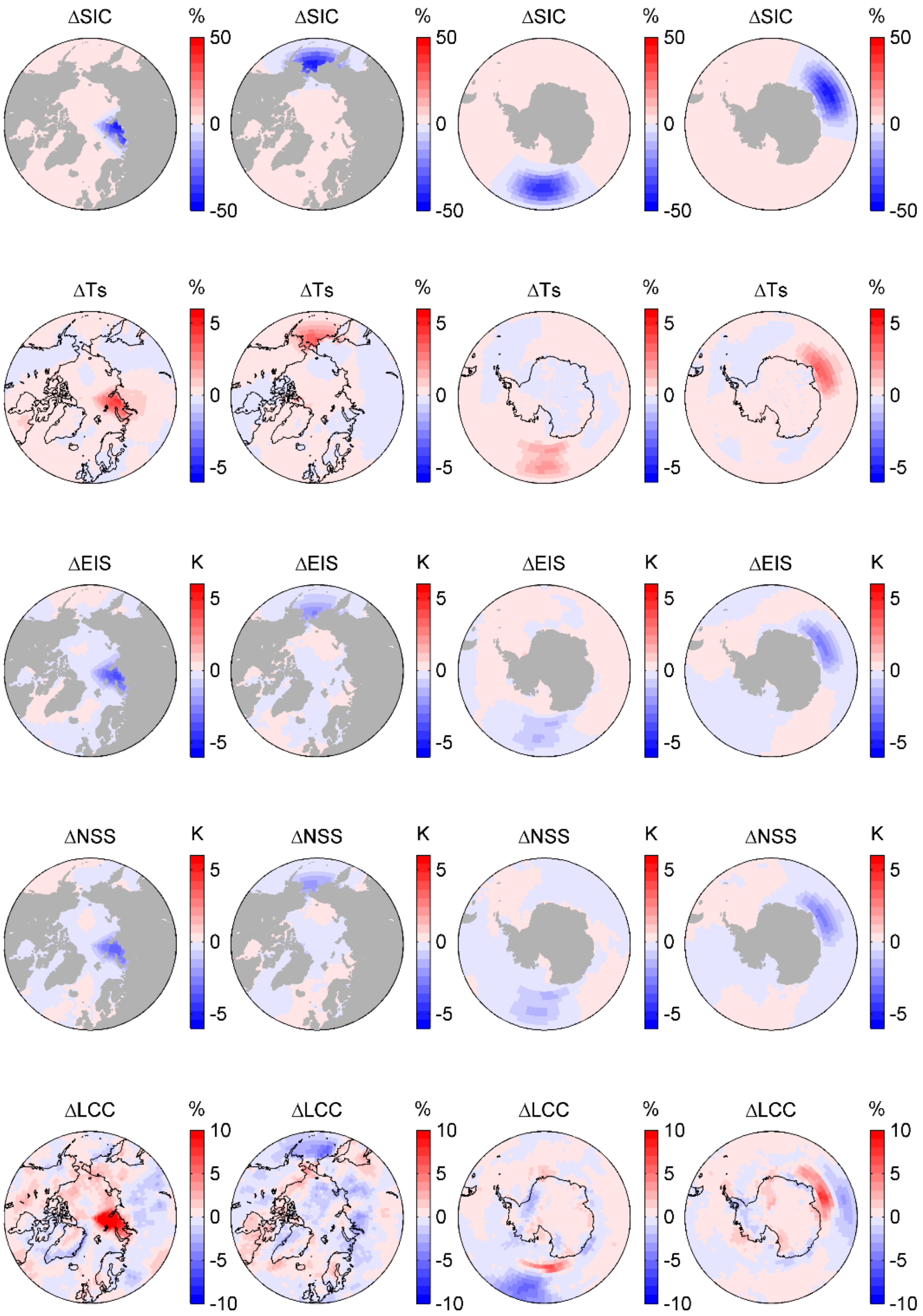

Fig. S8.

LCC responses to summertime (April-September in NH and October-March in SH) SIC reduction in four illustrative locations. The 4 columns denote results of 4 cases representing SIC changes in 4 illustrative patches. The upper row denotes the difference between SIC in the SIC decrease and SIC increase experiment. The second row denotes the change of surface temperature in response to the SIC change in each case. The third row denotes the change in EIS. The fourth row denotes change in near-surface static stability (NSS, calculated as the difference between potential temperature at 925hPa and 1000 hPa here). The bottom row denotes the change in LCC.

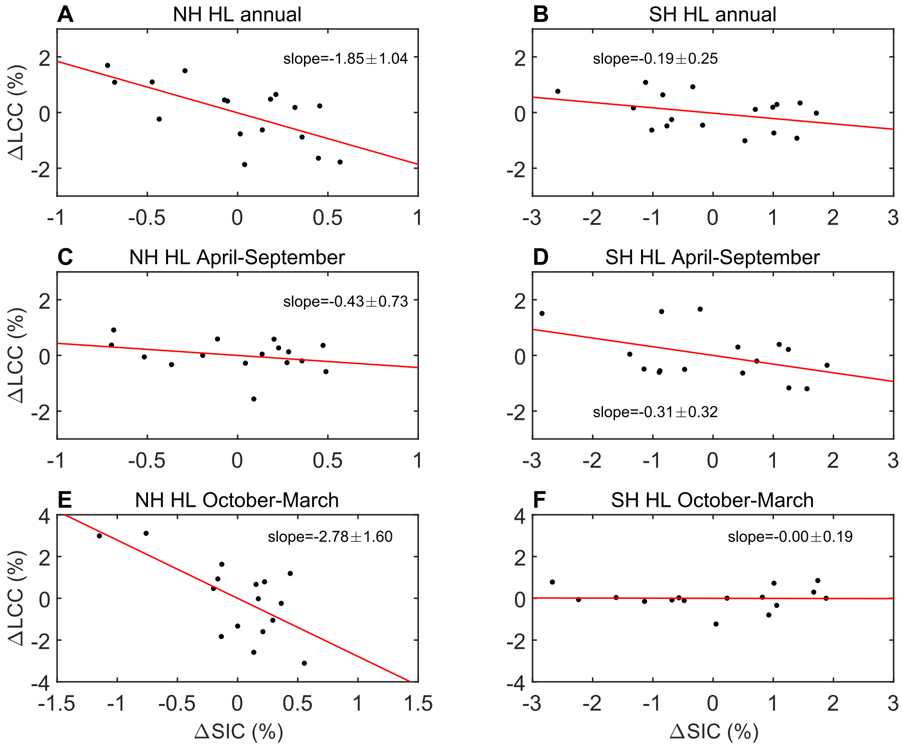

Fig. S9.

Relationship between average ΔSIC and ΔLCC in the Arctic (50°N-90°N) and Antarctic (50°S-90°S) regions during 2003-2019. SIC is calculated using AMIP SIC data, and LCC is calculated using MODIS level-3 data. SIC is negatively correlated with LCC in NH summer (C), but the correlation between LCC and SIC is zero in SH summer (F). The regression slope is shown in each panel, and the 95% uncertainty intervals are also displayed.

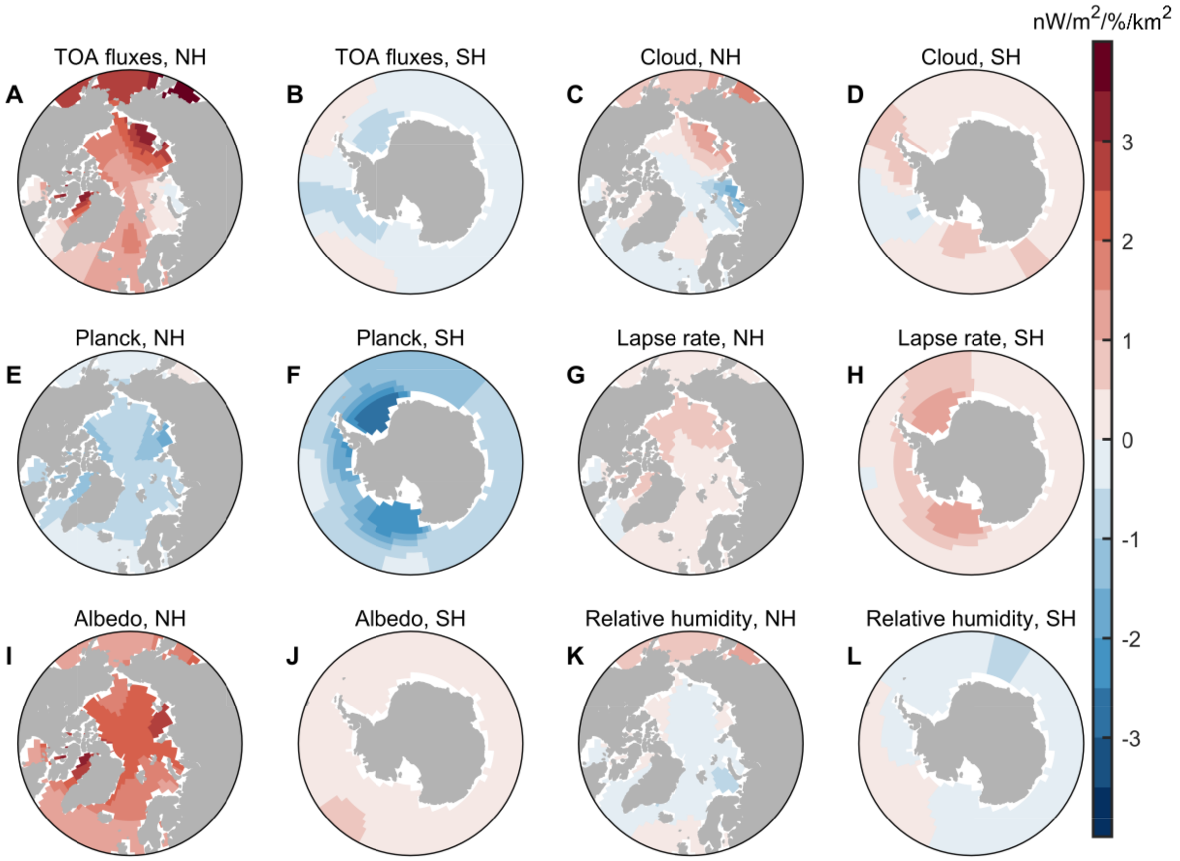

Fig. S10.

Same as Fig. 4, except for April-September.

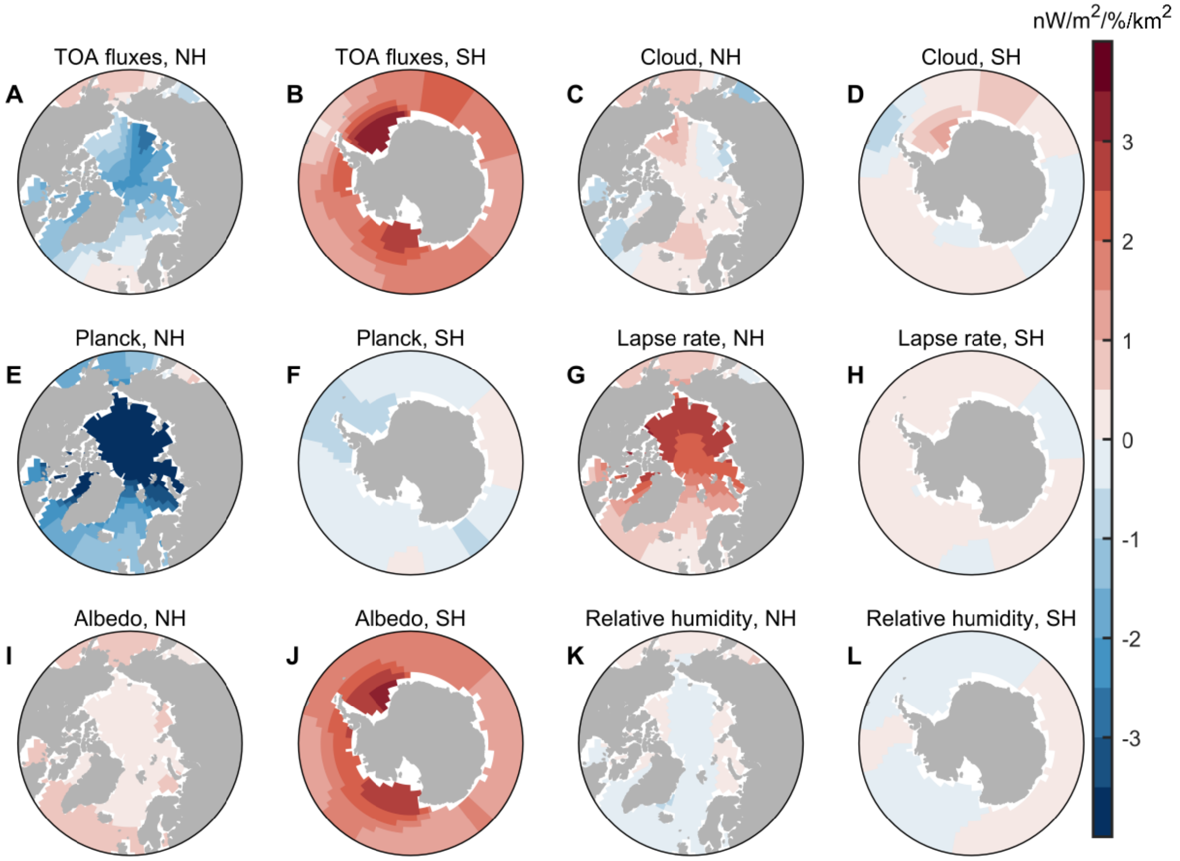

Fig. S11.

Same as Fig. 4, except for October-March.

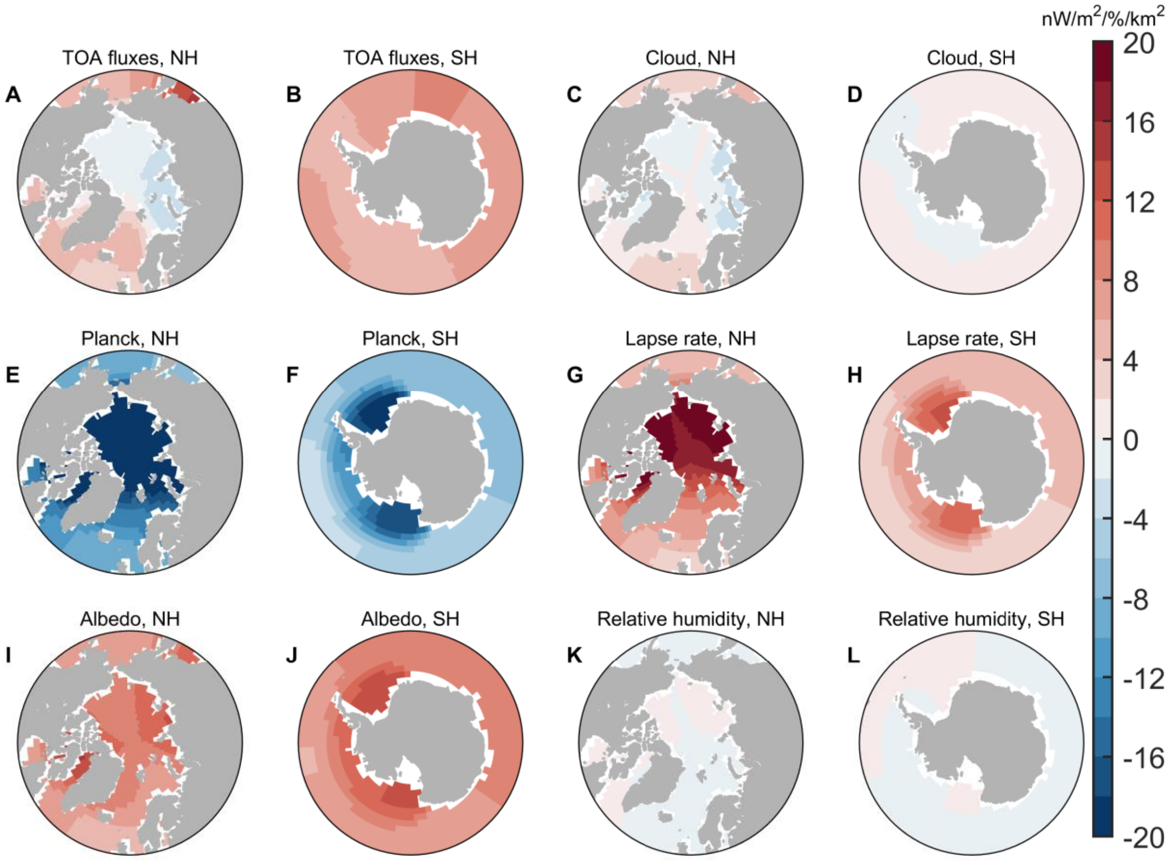

Fig. S12.

Same as Fig. 4, except for sensitivity of annual mean radiation in the high latitudes (50°S-90°S, 50°N-90°N) to regional SIC change.

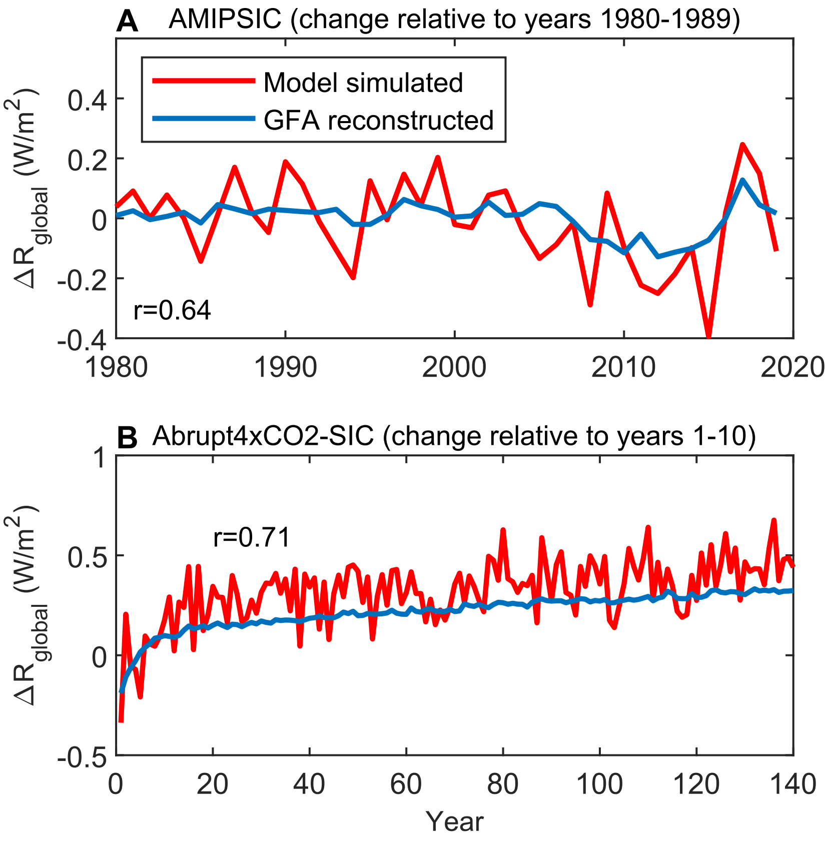

Fig. S13.

Reconstruction of SIC-induced radiation anomalies using the Green's function approach in the AMIPSIC experiment (A) and abrupt4xCO2-SIC experiment (B). The red lines denote actual values of ΔR, calculated as the change of R relative to the first 10 years (to reduce errors induced by non-SIC factors) of the corresponding experiment. The blue lines denote Green's function reconstructed values, i. e, ΔR reconstructed using the equation $\Delta R = \sum_i \frac{\partial R}{\partial SIC_i} * \Delta SIC_i + \varepsilon$, where ΔSIC_i is the annual SIC anomaly in a specific grid box. The correlation coefficient between reconstructed and actual values are shown in the figure. The Green's function approach underestimates the SIC-induced radiation change in abrupt4xCO2-SIC simulations due to nonlinearity, but the correlation is high (B), so it is valid for qualitative attribution analyses.

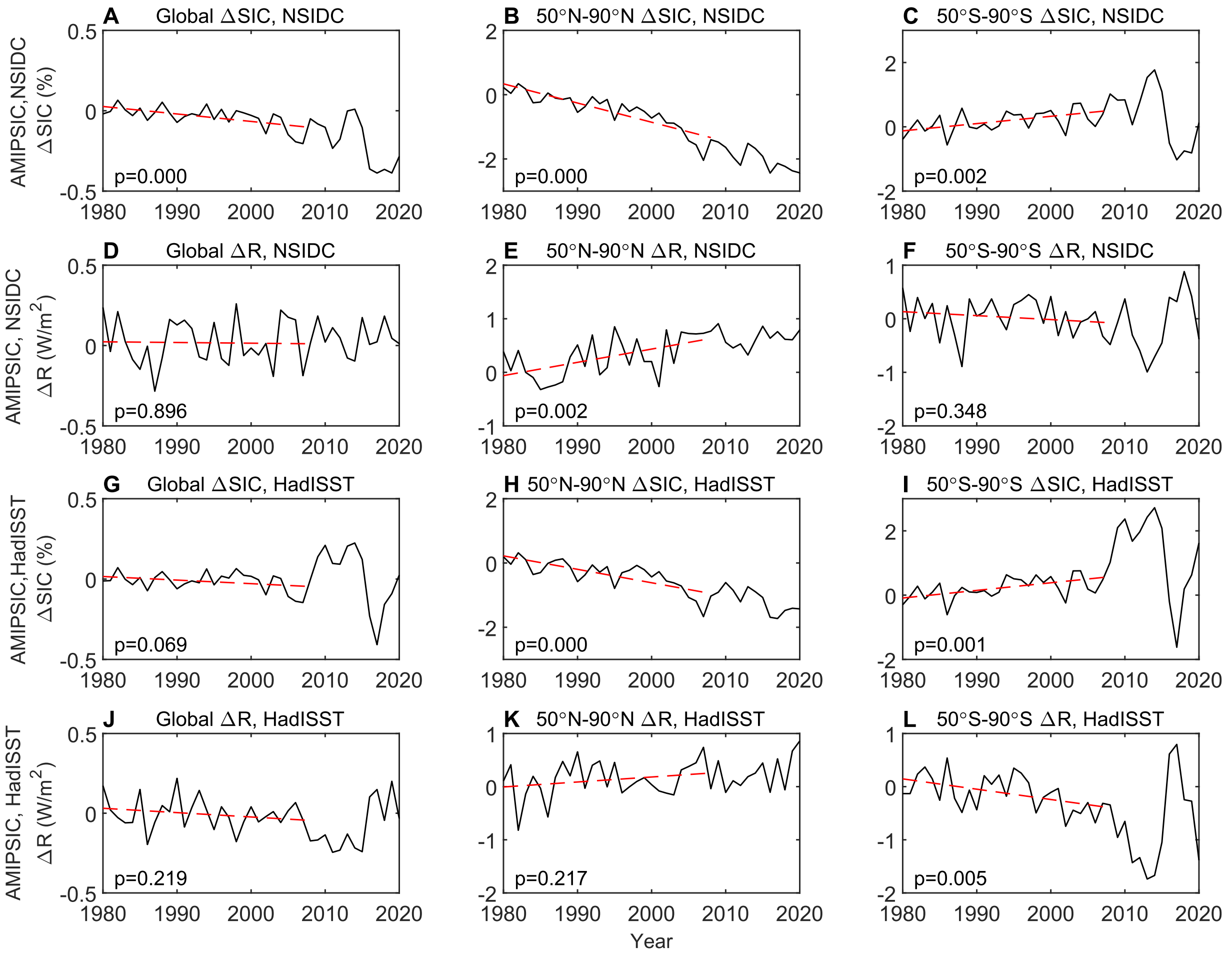

Fig. S14.

Same as Fig. 1, except for AMIPSIC experiments that are driven by different SIC datasets. (A-F) The experiment is driven by SIC from NSIDC, and the red dashed lines show the trends during 1980-2008, when the trends of ΔSIC and ΔR are both negative. (G-L) The experiment is driven by SIC from HadISST.

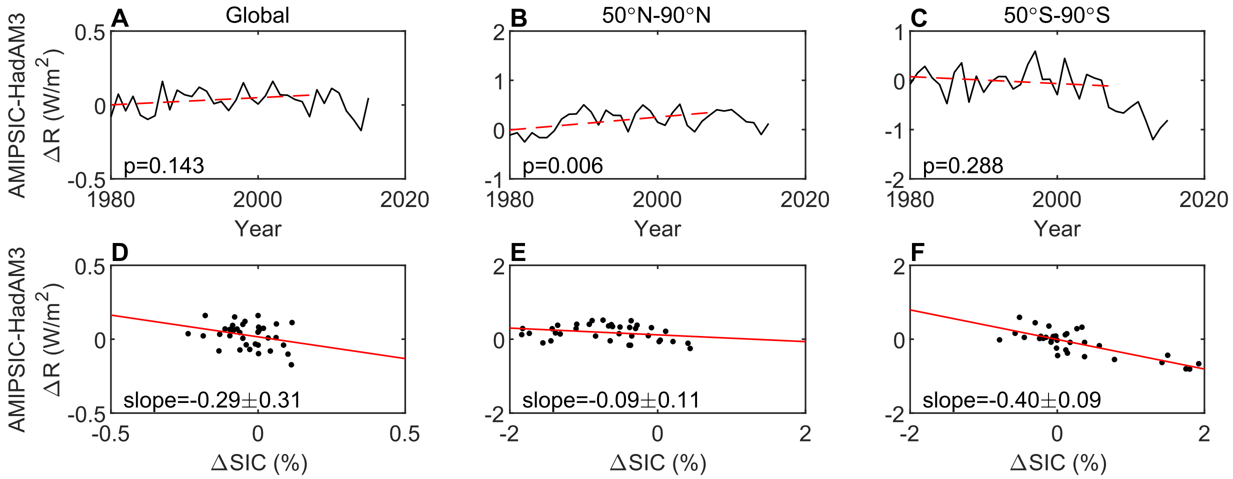

Fig. S15.

Relationship between Δ*SIC* and TOA radiation anomalies Δ*R* in AMIPSIC experiments performed with HadAM3. (A-C) are ensemble mean Δ*R*, which are similar as Figs. 1(J-L). (D-F) show the sensitivity of Δ*R* to Δ*SIC*, which are similar to the upper panels of fig. S5. The AMIPSIC-HadAM3 experiment is same as AMIPSIC experiment, except that there are 8 runs and each run covers the period of 1980-2015. Despite that the trend of ΔR is different in HadAM3, the sensitivity of Δ*R* to Δ*SIC* reduction is more than three times stronger in SH than that in NH (consistent to fig. S5), indicating that the SIC pattern effect characterized by hemispheric asymmetry in HadAM3 experiment is similar to that in CESM1.2.1 model.

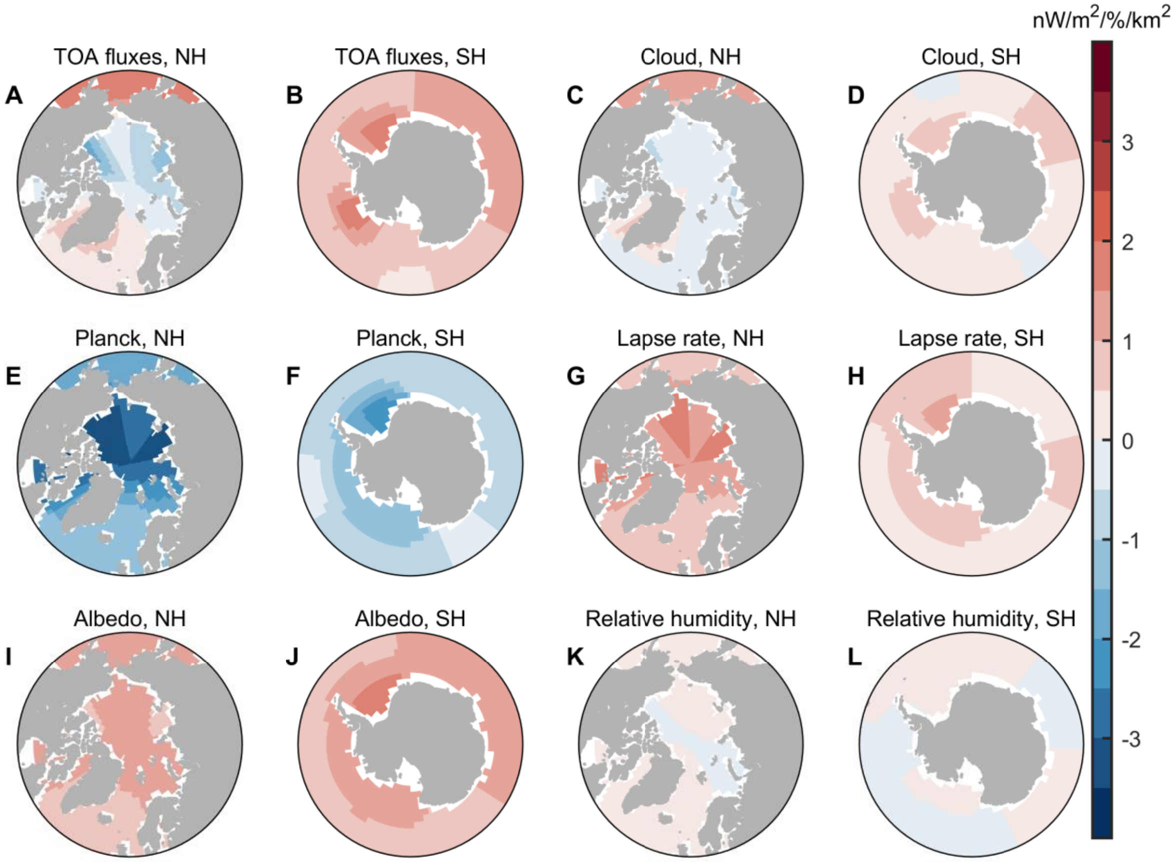

Fig. S16.

Same as Fig. 4, except for results calculated from SIC-SST covarying patch experiments, where SIC is same as the SIC patch experiments, but SST in each grid changes at the same time when SIC changes.

Table S1.

Idealized experiments performed with CESM1.2.1 that are used in this study.

Experiment Name	Description	Years run
AMIPFF	The forcings are fixed at preindustrial level, while SIC and SST are prescribed using historical data (*51*).	150 years (1870-2019) 3 runs for each experiment
AMIPSST	The forcings and SIC are fixed at preindustrial level (i. e., repeating climatological annual cycle), while SST are historical.	
AMIPSIC	The forcings and SST are fixed at preindustrial level, while SIC are historical (AMIP SIC, (*51*)).	
AMIPSIC with HadISST	Same as AMIPSIC, except that SIC are from HadISST dataset (*46*).	43 years (1979-2021) 3 runs for each experiment
AMIPSIC with NSIDC	Same as AMIPSIC, except that SIC are from NSIDC dataset (*47*).	
piControl (Coupled)	Preindustrial control run with active ocean model.	250 years
abrupt4xCO2 (Coupled)	The forcing is increased suddenly at Year 100 of PI-control run, and held constant.	150 years
abrupt4xCO2-SIC	The forcings and SST are fixed at PI-control level (i. e., repeating climatological annual cycle of SSTs derived from the model's piControl run), but the SIC are same as the abrupt4xCO2 experiment.	150 years, 3 runs
Control run for patch experiments	Prescribed SST/SIC experiment performed with present-day forcings, sea ice cover (SIC) and sea surface temperature (SST).	40 years
SIC patch experiments	The SIC of each target region is increased/decreased relative to the control run using Eq. (2), while SST is not changed. SIC outside the target region is not changed.	40 years for each of the 27x2 patch experiments
SIC-SST covarying patch experiments	The SIC of each target region is increased/decreased using Eq. (2), while the SST is changed simultaneously using Eq. (5).	40 years for each of the 27x2 patch experiments

Note: In the prescribed SST/SIC experiments, the sea ice thickness is set to be fixed in each hemisphere by the model. The sea ice thickness is calculated by the model in coupled runs.
